# Characterizing and Predicting the Resilient Modulus of Recycled Aggregates from Building Demolition Waste with Breakage-Induced Gradation Variation

**DOI:** 10.3390/ma15072670

**Published:** 2022-04-05

**Authors:** Yuanjie Xiao, Kunfeng Kong, Umar Faruk Aminu, Zhiyong Li, Qiang Li, Hongwei Zhu, Degou Cai

**Affiliations:** 1 School of Civil Engineering, Central South University, Changsha 410075, China; yjxiao@csu.edu.cn (Y.X.); kongkunfeng@csu.edu.cn (K.K.); umaraminu.ck@gmail.com (U.F.A.); 2 Ministry of Education Key Laboratory of Engineering Structures of Heavy Haul Railway, Central South University, Changsha 410075, China; 3 State Key Laboratory for High-Speed Railway Track Technology, China Academy of Railway Sciences Corporation Limited, Beijing 100081, China; zhuhongwei@yeah.net; 4 Hunan Communications Research Institute Co. Ltd., Changsha 410015, China; zhiyongli_jky@163.com; 5 College of Civil Engineering, Nanjing Forestry University, Nanjing 210037, China; liqiang2526@njfu.edu.cn

**Keywords:** building demolition waste, unbound granular materials, shear strength, resilient modulus, gradation, prediction model

## Abstract

Building demolition waste (BDW) has been massively stockpiled due to increasingly rapid urbanization and modernization. The use of recycled BDW as unbound granular base/subbase materials is among the sustainable, cost-effective, and environmentally friendly pavement construction alternatives. The resilient modulus is an important mechanical property of BDW-derived aggregates and mechanistic design input of pavements incorporating BDW. This paper presents the results of a comprehensive laboratory study on the shear strength and resilient modulus characteristics of BDW-derived aggregate materials. A series of monotonic triaxial compression tests and repeated-load triaxial (RLT) tests were conducted with five different gradations representing particle breakage and different stress paths. The apparent cohesion and internal friction angle of recycled BDW aggregates under consolidated drained conditions ranged from 35.3 to 57.5 kPa and from 30.2° to 54.3°, respectively. The apparent cohesion and internal friction angle also increased and decreased non-linearly with the increasing relative content of fine particles, respectively. The resilient modulus of recycled BDW aggregates gradually decreased with increasing relative content of fine particles at the same stress level. Both the deviator stress and confining pressure exhibited significant influences on the resilient modulus, while the effect of confining pressure was more profound. Based on laboratory testing data, a mechanistic-empirical model was developed to predict the resilient modulus of recycled BDW aggregates from gradation and stress-state variables. The findings could be useful for extended engineering applications of BDW in unbound granular pavement base/subbase construction.

## 1. Introduction

With the increasingly rapid urbanization and modernization around the globe, large quantities of building demolition waste (BDW) have been stockpiled, from which land use, public safety, and ecological environment concerns arise [1]. In recent years, the increasing awareness of environmental sustainability has demanded the substitution of these waste hazards for non-renewable natural resources in diverse construction industries. 

On average, less than 10% of stockpiled BDW is currently recycled for reuse in China and other developing countries, which is considerably lower than the recycling and reusing rate of more than 90% in developed countries [2]. On the other hand, the construction of transportation infrastructure, such as railroad tracks and roadway and airfield pavement, demands the use of stone-based granular materials in massive quantities, thus, causing the increasing depletion of non-renewable natural aggregate resources. Therefore, the efficient recycling and sustainable reusing of non-traditional construction and demolition wastes (CDWs), including BDWs have emerged as resource-conserving, environmentally friendly substituting solutions to alleviate such problems, especially in the new era of carbon peak and carbon neutrality (i.e., zero net-carbon emission) commitments [3,4].

CDWs are usually generated during the operations of construction, maintenance, repair, rehabilitation, and demolition of buildings and other infrastructures [5]. Featuring a heterogeneous nature, these wastes consist of distinct types of materials, such as concrete, bricks, ceramics, wood, glass, plastic, soil, and gypsum-based construction materials. Depending on the different geographical regions, natural environment, economic development, and construction practices, CDWs possess distinct compositions with bulky wastes (e.g., bricks and concrete) accounting for 70–80% of the total weight. 

Volodchenko et al. [6] confirmed the potential applicability of nonconventional argillous raw materials in the structurization optimization of building composites from X-ray diffraction and scanning electron microscopy analyses. Azevedo et al. [7] explored the potential use of the Curauá fibers in reinforcing wall-covering mortars, where the consistency, trapped water and air contents, compressive strength, water absorption and wet–dry durability were studied. Lu et al. [8] conducted a comprehensive study to evaluate the mechanical properties, functionalities, and environmental benefits of a new permeable material derived from recycled ceramic aggregates and bio-based polyurethane (PU) binder, and concluded that it could be used as the promising substitution for conventional Porous Asphalt (PA). 

Yu et al. [9] investigated the mechanical and engineering properties of asphalt mixtures modified with waste packing tape (WPT), e.g., the mixture workability, moisture susceptibility, modulus and strength, fatigue resistance, and rutting resistance. They found that the aforementioned performance of a WPT-modified mixture was greatly improved as compared to the raw asphalt mixture. Luhar et al. [10] and Mohajerani et al. [11] reviewed and confirmed the promising large-scale use of glass wastes for producing durable, cost-effective, and sustainable geopolymer concretes. Mishra et al. [12] studied the shear strength, California Bearing Ratio (CBR), and indirect tensile strength of subgrade soils stabilized with both recycled polyethylene terephthalate (PET) fibers and fly ash, and reported that the addition of such two waste materials of proper amounts led to improved strength parameters.

Aggregates recycled from bulky wastes (e.g., bricks and concrete) of CDWs have been used mainly in concrete production as constituents, including mortar, concrete, and bricks, as well as in pavement construction as marginal stone-based materials [13]. In the past few decades, extensive studies have been conducted to explore hydro-mechanical performances and assess the engineering suitability of stone-based granular materials derived from BDWs, such as recycled concrete aggregate (RCA), recycled clay masonry brick (RCM), and reclaimed asphalt pavement (RAP), in pavement construction. 

The results of triaxial tests conducted by Zhang et al. [14] showed that the recycled construction and demolition waste (CDW) exhibited much higher resilient moduli and lower accumulated permanent deformation as compared to embankment clay soils. This demonstrated that the substitution of recycled wastes for clay soils would improve the structural capacity and reduce rutting damage. Silva et al. [15] presented a review of the most important physical properties of different types of recycled aggregates (RAs) and their comparisons with natural aggregates (NAs) as well as of how these properties affect their hydro-mechanical behavior when compacted. 

The results collected from the literature indicate that the engineering performance of most RAs is comparable to that of NAs and can be used in unbound pavement layers or in other applications requiring compaction. Vieira et al. [16] presented a laboratory study performed on two coarse-grained recycled aggregates and discussed their suitability for constructing embankments and capping layers of transport infrastructures, including rural and forest roads. The results showed that the mixed CDW aggregates possessed suitable characteristics for potential use in the construction of embankments, as well as for serving as alternative aggregates for the subbase layers of rural and forest roads. 

In addition, Vieira [17] assessed, using direct shear and pullout tests, the possibility of using fine-grained materials recycled from CDW as backfills of geosynthetic-reinforced structures to replace the natural soils otherwise typically used in the construction of these structures. Those studies reported that, even though recycled BDW aggregates contain different combinations of complex constituents, such as asphalt concrete, cement concrete, brick, and glass, their strength and deformation properties are relatively comparable to those of traditional virgin aggregates [18,19]. 

Saberian et al. [20] conducted a series of large-scale direct shear tests (LDST) to evaluate the shear strength properties of RCA mixed with crumb rubber (derived from used tires) of different sizes and amounts and found that such mixtures of RCA and crumb rubber exhibited an increase in the apparent cohesion due to the addition of fine or coarse crumb rubber and satisfied the shear strength requirements for potential applications in a pavement base/subbase. Arulrajah et al. [21] conducted direct shear tests and consolidated drained triaxial tests to characterize the shear strength properties of recycled BDW materials. They further classified their shear responses into two different categories, i.e., the dilatancy-induced peak strength and dilatancy-associated strain-hardening behaviors. 

According to their testing results, RCA and RCM clearly exhibited dilatancy-induced peak strength preceded by the occurrence of the maximum dilatancy ratio, whereas higher dilatancy ratios were associated with greater peak friction angles; on the other hand, RAP showed dilatancy-associated strain-hardening behavior even with a relatively high magnitude of dilatancy. From those studies focusing on the shear strength characteristics of compacted BDW materials, it can be inferred that they have the potential to be used in pavement base/subbase applications as they meet the requirements of the minimum effective friction angles required.

As an important mechanical performance indicator, the resilient modulus (*Mr*) characterizes the ability of compacted unbound granular pavement materials to resist elastic deformation induced by repeated traffic loading. The resilient modulus has a direct impact on pavement performance, such as fatigue cracking and rutting resistance, and thus has become a key input parameter in the current mechanistic pavement analysis and design [22,23,24]. The resilient behavior of traditional aggregates under different working conditions and material compositions has been a well-studied topic. Diagne et al. [25] studied the performance of RCM and RCA blended in different proportions as a fill material of a granule base, and found that the resilient modulus of this fill material decreased with the increasing moisture content and RCM content. 

Silva et al. [6] conducted a literature survey to compare the physical and mechanical properties of recycled CDW aggregates and natural aggregates (NA) and indicated that the material composition and the degree of aggregate particle breakage affected the resilient modulus. Arisha et al. [26] studied the resilient modulus of recycled CDW fill materials with different contents of recycled bricks by following the AASHTO-T307 testing protocol. They found that the resilient modulus exhibited a downward trend with increasing content of recycled bricks, and they proposed a resilient modulus prediction model by incorporating the content of recycled bricks. 

Soleimanbeigi et al. [27] evaluated the resilient modulus of RAP samples. According to their testing results, the resilient modulus of RAP samples was greater than that of RCA or NA samples at 20–30 °C; however, when the temperature was raised to 50 °C, the resilient modulus of RAP samples decreased significantly, whereas that of RCA or NA samples remained basically unchanged. Recycled CDW aggregates are prone to particle breakage and degradation, especially under the application of heavy traffic loading, harsh environmental loading (e.g., wet–dry/freeze–thaw cycles), or both. Potentially marked gradation variations could then occur throughout their service life periods and detrimentally affect their hydro-mechanical behavior and in-service performance. However, few previous studies have addressed such gradation variations of recycled CDW aggregates and their possible consequences, which may hinder their reliable and widespread use. It is then of great significance and necessity to perform an in-depth study in this regard.

In light of the above-mentioned research need, this study aims to investigate the feasibility and applicability of recycled BDW materials as unbound granular fill materials for a pavement base/subbase. Five different gradations were designed to represent particle breakage-induced gradation variations. A series of monotonic triaxial compression tests at consolidated drained conditions and repeated-load triaxial (RLT) tests were conducted to investigate the shear strength and resilient modulus characteristics of recycled BDW aggregates with five different gradations. Based on the laboratory testing data obtained, a mechanistic-empirical prediction model of the resilient modulus was then proposed by incorporating gradation variations and stress states. We expect that the findings of this study will be able to provide technical reference and guidance for promoting the use of recycled BDW as unbound granular materials in pavement base/subbase applications.

## 2. Materials and Methods

### 2.1. Materials Tested

**Figure 1:**
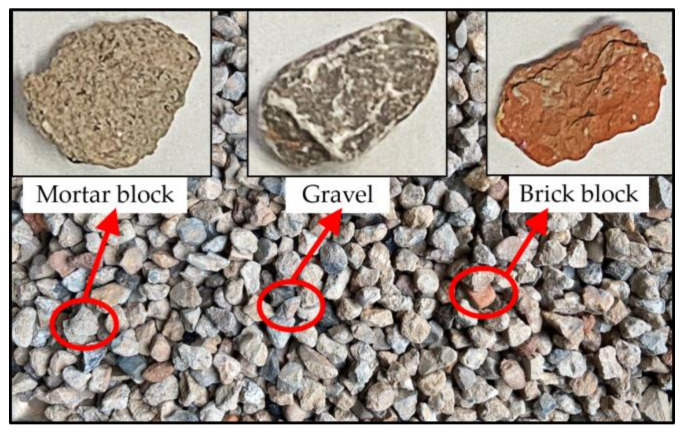
Aggregate particles recycled from BDW.

The materials tested in this study were recycled BDW aggregates that were generated from different building demolition sites in Changsha, China and then transported to a processing center for crushing, screening, and stockpiling. As illustrated in Figure 1, the recycled BDW aggregates mainly consisted of used aggregate, cement mortar, and red brick particles, of which the combined percentage by mass was more than 95%. The recycled BDW aggregate particles were divided into four size groups: 0–5 mm, 5–10 mm, 10–20 mm, and 20–40 mm. The individual percentage by mass of each of the three different aggregate types (i.e., used aggregate, cement mortar, and red brick) within each of the four different size ranges was obtained by random sampling and manual sorting as listed in Table 1. 

**Figure 2:**
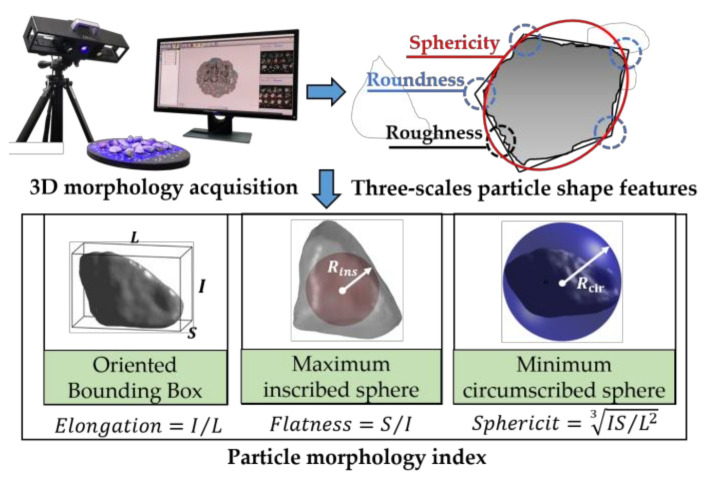
Illustration of the shape quantification of coarse particles recycled from BDW.

The morphological characteristics of coarse particles significantly affect the formation of the load-transferring aggregate skeleton structure and thus key hydro-mechanical performance (e.g., the resilient modulus, shear strength, permanent deformation, and permeability) of unbound aggregates recycled from CDW [28]; therefore, it is necessary to characterize and quantify the morphology of individual coarse particles, i.e., form, angularity, and surface texture. A total of 540 particles were randomly selected to represent the three different types of recycled BDW aggregates (i.e., used aggregate, cement mortar, and red brick) within four different size ranges. Those selected particles were then processed by a three-dimensional (3D) laser scanner for shape acquisition, and their 3D models were reconstructed accordingly for calculating the shape indices, including elongation, flatness, and sphericity [29] as shown in Figure 2.

**Figure 3:**
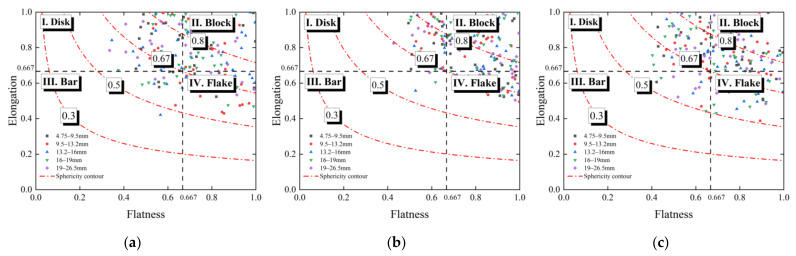
The shape classification results of three different types of aggregate particles recycled from BDW. (**a**) Used aggregate particles. (**b**) Cement mortar particles. (**c**) Brick particles.

Zingg’s bivariate method [29] was used to classify the shape of different types of coarse BDW particles, and the classification results are shown in Figure 3. As can be seen from Figure 3, most of the coarse BDW particles resemble blocks or disks, and most of the sphericity values of used aggregate and brick particles range from 0.5 to 1; on the other hand, the shape distribution of cement mortar particles is relatively more concentrated, i.e., there are almost no bar-shaped particles, and their sphericity values range almost from 0.67 to 1. 

In addition, there appeared to be no significant difference in the particle morphology among different particle size groups of the same type of recycled BDW aggregates. Therefore, the subsequent laboratory triaxial tests in this study were conducted by controlling the particle shape difference so that the calculated particle shape indices of different specimens remained relatively similar or close to each other; in other words, particle shape was excluded from the list of explanatory variables considered in the testing matrix of this study and will be the subject of future studies.

The basic index properties of unbound aggregate materials derived from recycled BDW aggregates, such as the Atterberg limits, crushing value, water absorption, and specific gravity, were measured by following related testing standards or specifications [30,31], as listed in Table 2. It can be seen from Table 2 that the recycled BDW aggregates possessed a relatively high crushing value and water absorption due to the presence of aged cement mortar fully or partially covering aggregate particles and small cracks on the surface of recycled aggregates. Therefore, recycled BDW aggregates are likely to be more susceptible to particle crushing (or breakage) and moisture-related degradation when compared to virgin aggregates, which may eventually cause differences in the mechanical performance and long-term durability between these two different types of materials.

### 2.2. Gradations Designed

The gradation itself has a crucial influence on the hydro-mechanical properties of unbound granular materials, including the resilient modulus, shear strength, permanent deformation, permeability, and durability. In this study, the gravel-to-sand ratio (*G/S*) parameter, which was proposed based on the theory of particle packing, was adopted for the gradation design and control of laboratory triaxial specimens. The *G/S* parameter and its applicability to traditional and reclaimed unbound granular materials have been verified and supported in the literature [32,33,34]. To be specific, the *G/S* parameter can be calculated from the maximum particle size $D_{\max}$ and the shape parameter *n* of the gradation curve according to Equation (1), wherein the shape parameter *n* of the gradation curve can be regressed from the mechanical sieve analysis result according to Equation (2).

(1) $$G/S=\frac{1-p_{4.75}}{p_{4.75}-p_{0.075}}=\frac{D_{\max}{}^{n}-4.75^{n}}{4.75^{n}-0.075^{n}}$$

(2) $$P_{d}={\left(\frac{d_{i}}{D_{\max}}\right)}^{n}$$

where $P_{d}$ is the percentage of materials passing the sieve with an opening size of $d_{i}$; $d_{i}$ is the sieve opening size (mm); $D_{\max}$ is the maximum particle size (mm); and *n* is the parameter controlling the shape of gradation curves.

**Figure 4:**
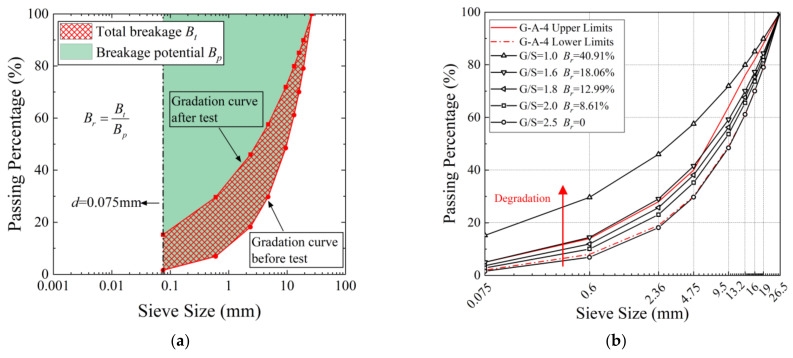
Gradation curves of recycled BDW aggregates representing particle-breakage-induced gradation variations. (**a**) Definition of particle breakage index *B_r_*. (**b**) Gradation curves and their corresponding *B_r_*.

The recycled BDW aggregates used in the laboratory testing program can be classified as well-graded gravel (GW). They were oven-dried and sieved apart into different particle size groups. The maximum particle size was set as 26.5 mm due to the specimen size limit of the triaxial testing apparatus, and particles larger than 26.5 mm were picked out and replaced by smaller particles of equal weights. The specimens of the recycled BDW aggregates were prepared according to the five different gradations shown in Figure 4b of which the *G/S* values were 1.0, 1.6, 1.8, 2.0, and 2.5, respectively. 

Note that the upper-limit and lower-limit gradation curves denote the G-A-4 gradation band for high-grade highway pavement base/subbase materials as recommended in China’s Technical Guidelines for Construction of Highway Roadbases (JTG/TF20-2015). Quantitatively defining the degree of particle breakage is necessary. The effective way is to use the particle size distribution curves measured before and after tests to estimate the amount of particle crushing [35]. A relative breakage index *B_r_* proposed by Hardin [35] and shown in Figure 4a was adopted in the current work, which can be expressed as:

(3) $$B_{r}=\frac{B_{t}}{B_{p}}$$

where *B_p_* is called the crushing potential, i.e., the area between the initial grading (gradation curve before test) and boundary of 0.075 mm; and the parameter *B_t_* is called the total crushing, i.e., the area between the initial grading and current grading (gradation curve after test). According to this definition, the gradation of *G/S* = 2.5 is considered as the initial state, and then the particle breakage index corresponding to the other four gradations (*G/S* = 2.0, 1.8, 1.6, and 1.0) can be calculated separately. 

**Figure 5:**
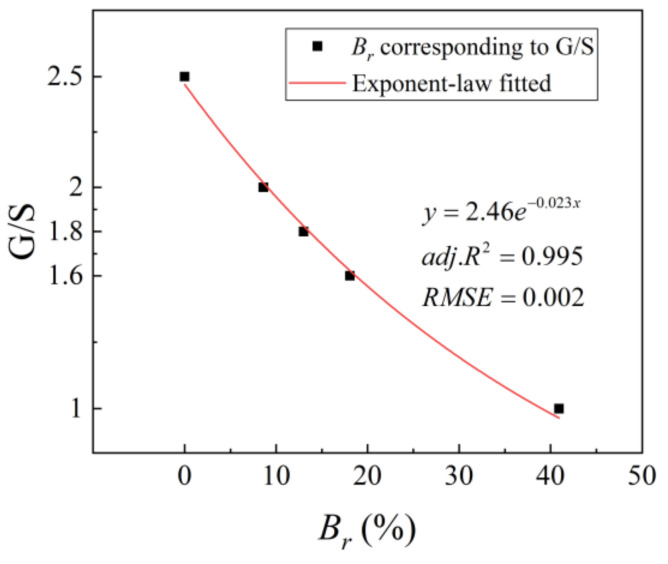
Fitting results of the particle breakage index values *B_r_* against the G/S parameter.

These five different gradations were chosen to represent the different stages of particle breakage of recycled BDW aggregates as indicated by the particle breakage index values *B_r_* in Figure 4b. In other words, as the value of the parameter G/S decreases, the relative content of fine particles gradually increases, which indicates a process of degradation. This phenomenon is expressed in the particle size distribution curve as a shift of the gradation curve to the upper left. The correspondence between the gradation parameter G/S and the breakage index values *B_r_* is given in Figure 5 and Equation (4).

(4) $$G/S=2.46e^{-0.023x}$$

### 2.3. Description of Laboratory Testing Program

#### 2.3.1. Compaction Tests

Laboratory compaction tests were conducted according to the following Chinese standards (JTG 3430-2020) [30] with the related compaction parameters listed in Table 3, from which the compaction curves of the gravitational moisture content versus achieved dry density were obtained to determine the optimal moisture content (OMC) and maximum dry density (MDD) for each of the five different gradations. Specifically, a cylindrical mold with an inner diameter of 152 mm and an effective height of 120 mm was used for the laboratory compaction tests. The recycled BDW aggregates prepared for compaction tests according to the designated gradations and moisture contents were placed into the mold in three sub-layers, and each sub-layer was then compacted by 98 blows of a 4.5-kg hammer falling freely from a height of 450 mm. This experimental method of compaction is equivalent to the Modified Proctor’s compaction (AASHTO T180), and the hitting-work per unit is 2691 KJ/cm^3^.

#### 2.3.2. Monotonic Triaxial Compression Tests

**Figure 6:**
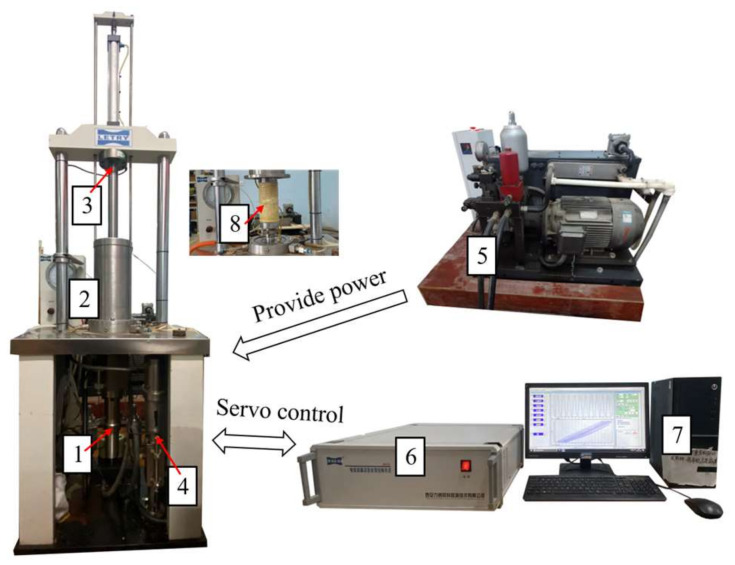
The laboratory triaxial apparatus for monotonic and repeated-loading tests. 1. Vertical loading system, including a hydraulic servo actuator and transducer for axial deformation; 2. triaxial cell (specimens); 3. transducer for axial force; 4. confining pressure servo; 5. oil pressure system, including a high-pressure syringe pump; 6. controller; 7. computer interface system; and 8. specimen.

The monotonic triaxial compression tests under consolidated drained (CD) conditions (herein abbreviated as CD triaxial tests) were performed in the laboratory to determine the shear strength properties of recycled BDW aggregates. The use of consolidated drained tests was meant to simulate long-term field conditions. The CD triaxial tests were performed using an automated apparatus shown in Figure 6 and following the testing protocol T0146 specified in the Chinese standard (JTG 3430-2020). 

The specimens were fabricated in a split mold with a 100-mm inner diameter and 200-mm height. According to the optimal moisture content and maximum dry density determined from the compaction tests, the specimens were first prepared at the optimal moisture content, placed into the split mold in five sub-layers, and then compacted to achieve the relative compaction (the ratio of achieved dry density to maximum dry density) of 95%. The specimens were covered by special latex membranes of 0.5 mm thickness to refrain from collapse during the specimen preparation and loading stages. 

The isotropic consolidation stage was conducted with three different levels of effective confining pressure, i.e., 50, 100, and 200 kPa. The specimens were then consolidated under drained conditions. The subsequent monotonic compression stage (or shearing stage) was performed at the strain control mode with the pre-selected loading rate of 1 mm per minute. The criterion adopted in the study for terminating the tests was the occurrence of either peak axial (or deviator) stress or the axial strain of 15%, whichever took place first. For the latter case, the axial stress corresponding to the axial strain of 10% was treated as the peak.

#### 2.3.3. Repeated Load Triaxial Tests

The specimen preparation procedure of the repeated-load triaxial (RLT) tests for characterizing the resilient modulus behavior was the same as that for the aforementioned monotonic triaxial compression tests of five different gradations of specimens at optimum moisture and 95% maximum dry density. In this study, the AASHTO-T307 testing protocol was adopted for the RLT tests, i.e., the entire loading process consisted of the initial pre-loading conditioning stage and the subsequent applications of a total of 15 different sequences of repeated loading [36]. 

**Figure 7:**
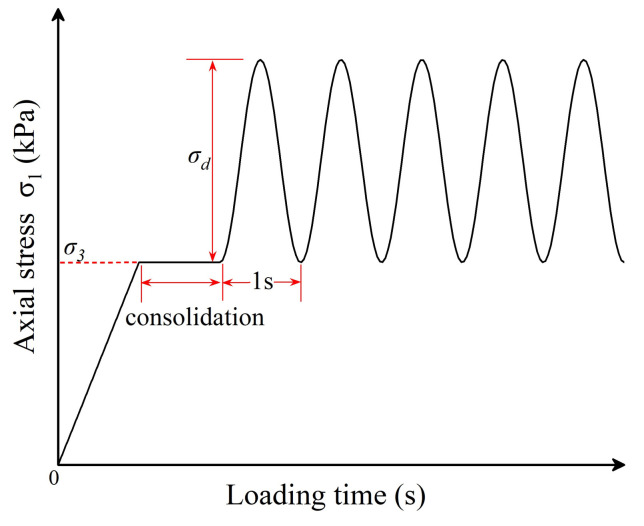
The time–history curve of axial stress applied during the laboratory repeated-load triaxial tests.

The different combinations of confining pressure and deviator stress specified for the conditioning stage and the repeated-loading sequences are summarized in Table 4. The time–history curve of axial stress (*σ*_1_) applied is shown in Figure 7. The loading waveform is a half-sine wave with a frequency of 1 Hz. The triaxial specimens were conditioned for 1000 load applications with the combination of 103.4 kPa confining pressure and 103.4 kPa deviator stress in order to eliminate contact deformation between the loading cap (or base) and the specimens, followed by the 15 different sequences of repeated loading. 

**Figure 8:**
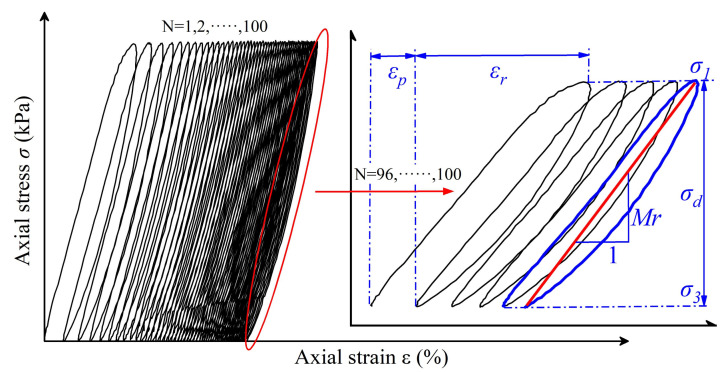
Schematic diagram of the resilient modulus defined from the axial stress–axial strain loops.

Each of the 15 sequences included 100 repeated load applications. If the accumulated plastic axial strain of the specimens exceeded 5% during loading, the tests were then terminated. The resilient modulus corresponding to each of the last five load applications of each repeated-loading sequence was calculated via Equation (5) as illustrated in Figure 8. The average value of the resilient moduli of the last five load applications was taken as the representative value of the resilient moduli of each sequence of repeated loading (or equivalently each combination of stress states).

(5) $$M_{r}=\sigma_{d}/\varepsilon_{r}$$

where $M_{r}$ is the resilient modulus (MPa); $\sigma_{d}$ is the deviatoric stress (MPa); and $\varepsilon_{r}$ is the resilient (or recoverable) axial strain.

## 3. Testing Results and Analysis

### 3.1. Laboratory Compaction Results

The laboratory compaction test results (i.e., the OMC and MDD) are shown in Table 5. Those OMC and MDD values corresponding to five different gradations were used subsequently for preparing specimens of monotonic triaxial compression tests for the shear strength properties and repeated-load triaxial tests for the resilient modulus.

### 3.2. Shear Strength Properties

**Figure 9:**
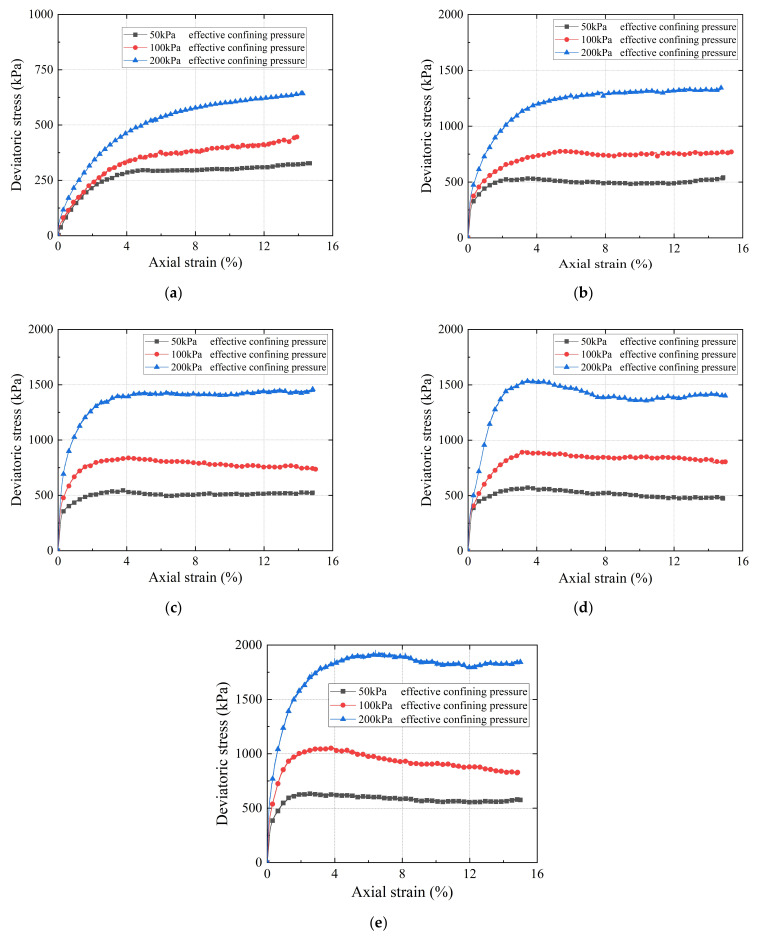
Deviator stress versus axial strain curves determined from the consolidated drained monotonic triaxial compression tests for the recycled BDW aggregates with different gradations: (**a**) G/S = 1.0; (**b**) G/S = 1.6; (**c**) G/S = 1.8; (**d**) G/S = 2.0; and (**e**) G/S = 2.5.

The deviator stress versus axial strain curves of the specimens with five gradations were obtained at three different levels of confining pressure, i.e., 50, 100, and 200 kPa, as plotted in Figure 9. It can be seen that, as the G/S value gradually decreases, i.e., the degree of degradation continues to increase, the peak deviatoric stress at the same confining pressure also gradually decreases. From each of the curves in Figure 9, the peak deviatoric stress at failure under each confining pressure was determined. 

**Figure 10:**
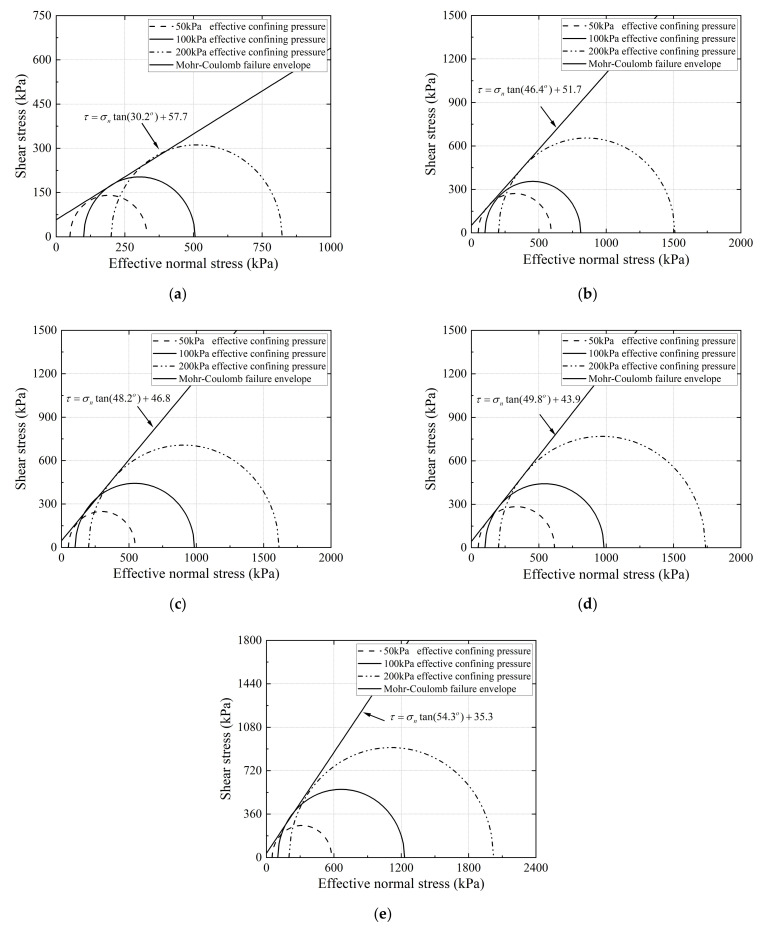
Mohr–Coulomb failure envelopes determined from the consolidated drained monotonic triaxial compression tests for recycled BDW aggregates with different gradations: (**a**) G/S = 1.0; (**b**) G/S = 1.6; (**c**) G/S = 1.8; (**d**) G/S = 2.0; and (**e**) G/S = 2.5.

The Mohr–Coulomb envelopes fitted from the results of monotonic triaxial compression tests are shown in Figure 10. Accordingly, the shear-strength parameters, i.e., the internal friction angle and apparent cohesion, were then calculated for each of the five different gradations as listed in Table 5. It can be seen that, as the relative content of fine particles in the specimens increases, i.e., the gradation parameter *G/S* value decreases, the apparent cohesion gradually increases, and the internal friction angle gradually decreases. This indicates that the degradation or particle breakage weakens the frictional interaction among the particles inside the specimen, resulting in a reduction in overall strength.

### 3.3. Resilient Modulus Properties

#### 3.3.1. Hysteresis Curves of Deviator Stress versus Axial Strain

**Figure 11:**
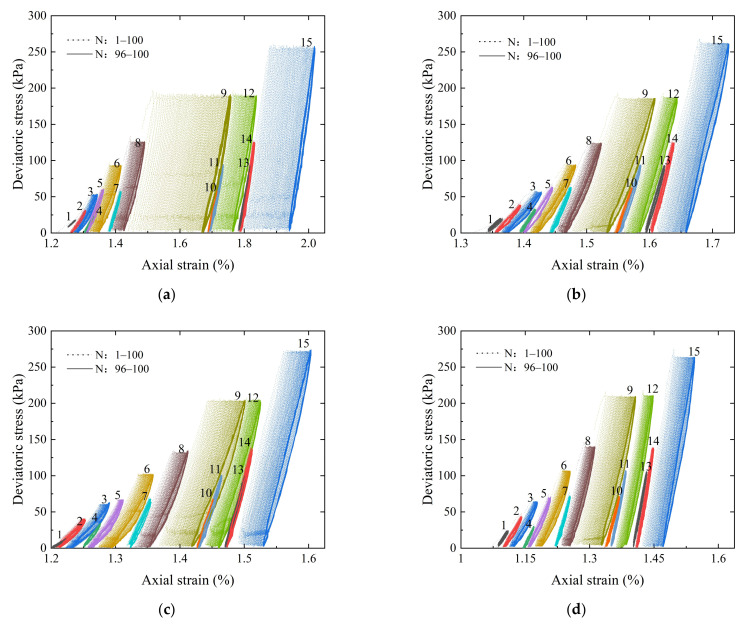

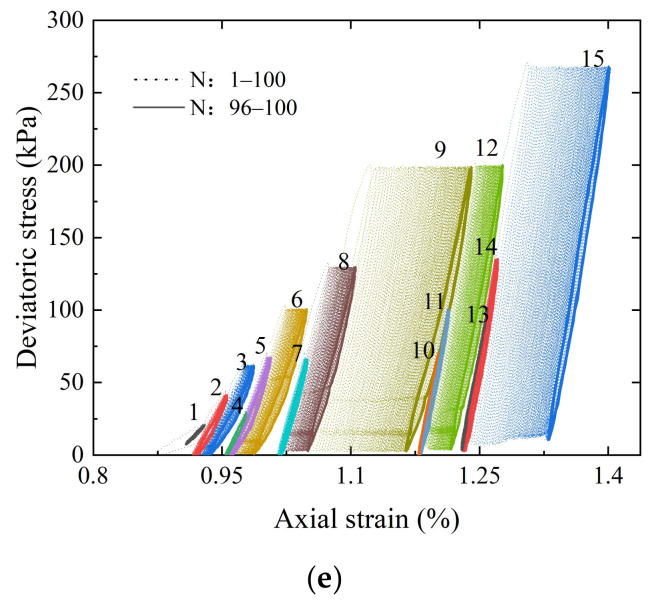
Hysteresis curves of the deviator stress versus axial strain of recycled BDW aggregates with different gradations: (**a**) G/S = 1.0; (**b**) G/S = 1.6; (**c**) G/S = 1.8; (**d**) G/S = 2.0; and (**e**) G/S = 2.5.

Figure 11 plots the hysteresis curves of the deviator stress versus axial strain for recycled BDW aggregate specimens with different gradations during the applications of 15 different loading sequences. Each of the 15 groups of dashed lines represents the complete number of 100 repeated-load applications of each loading sequence, while each of the 15 groups of solid lines represents the last five repeated-load applications (i.e., the 96th to 100th load applications) within each loading sequence. 

It can be observed from Figure 11 that, during each loading sequence of confining pressure and deviator stress, the hysteresis loops of deviator stress versus axial strain tend to overlap as the number of repeated-load applications gradually increases. This indicates that the stability of internal structures of the specimens increases gradually. Further, the axial deformation tends to gradually become completely elastic, i.e., the plastic axial strain gradually becomes stable and ceases to further accumulate. 

The final accumulated plastic axial strain values of all the specimens met the specification requirements (i.e., less than 5%). In addition, as the confining pressure increased, the slope of each hysteresis loop of deviator stress versus axial strain tended to “bend” towards the left side; in contrast, the deviator stress level had no obvious effect on the slopes of the hysteresis loops. It can be further seen from Figure 11 that the final accumulated plastic axial strain of the specimens increased with decreasing G/S value (or degradation developed) and that the slope of the hysteresis loop decreased gradually.

#### 3.3.2. Effects of the Stress State on the Resilient Modulus

**Figure 12:**
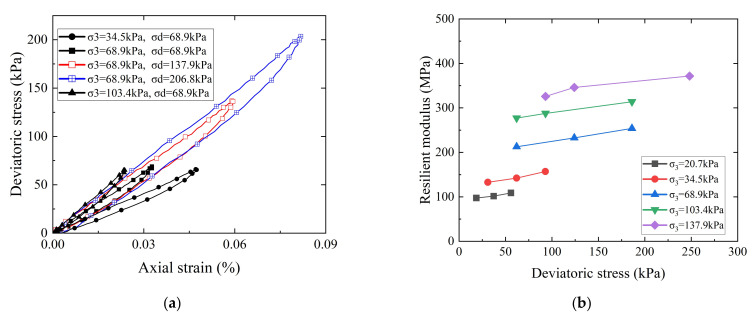
Effects of different stress states on the resilient modulus of recycled BDW aggregates: (**a**) Hysteresis curves of the deviator stress versus axial strain. (**b**) The resilient modulus versus deviator stress.

Figure 12a shows the hysteresis curves of the deviator stress versus axial strain of recycled BDW aggregates with a G/S value of 1.8 under different combinations of confining pressure and deviator stress (or different stress states). It can be seen that the hysteresis curves corresponding to the same level of deviator stress (e.g., 68.9 kPa) clearly exhibit an increasingly downward trend with decreasing confining pressure, thus, indicating that the resilient modulus of the specimen decreases gradually. Figure 12b shows the curves of the resilient modulus (*Mr*) versus deviator stress under different levels of confining pressure for the recycled BDW aggregate specimens with G/S value of 1.8. 

It can be seen from Figure 12b that the *Mr* increases by 11.6–19.4% with increasing deviator stress under the same level of confining pressure; on the other hand, the *Mr* increases by up to 95.1% with increasing confining pressure under the same level of deviatoric stress (e.g., 68.9 kPa). Therefore, both the deviator stress and confining pressure exert great influences on the *Mr* of recycled BDW aggregates, and the influence of the confining pressure is relatively greater than that of deviator stress. The reason is likely that the friction strength of coarse particles plays a leading role compared to the cohesion strength, and that the strengthening effect of confining pressure on the resilient modulus and stiffness of coarse-grained soils is much greater than the strain-hardening effect of shear displacement caused by increasing deviator stress on the internal structure of coarse-grained soils.

#### 3.3.3. Effect of Gradation on the Resilient Modulus

**Figure 13:**
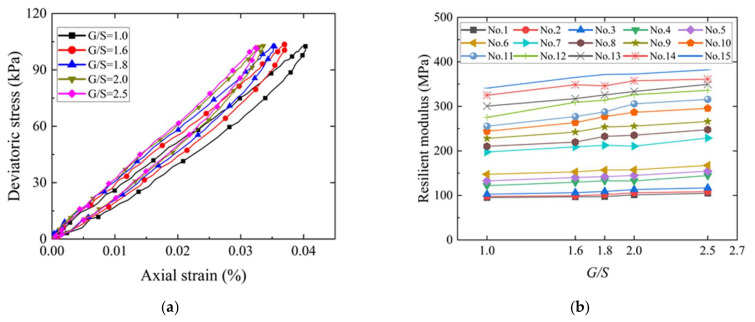
Effect of different gradations (G/S values) on the resilience modulus of unbound granular materials recycled from CDW: (**a**) Hysteresis curves of the deviator stress versus axial strain. (**b**) The resilient modulus versus G/S.

Figure 13a shows the hysteresis curves of the deviator stress versus axial strain of recycled BDW aggregates with different G/S values under the conditions of optimal moisture content and 95% maximum dry density (i.e., a relative compaction of 95%), where both the confining pressure and deviator stress are 103.4 kPa. It can be seen from Figure 13a that, under the same stress state, the *Mr* of recycled BDW aggregates decreased with the increasing relative content of fine particles (i.e., decreasing G/S value) as indicated by the gradually decreasing slope of hysteresis loops. Figure 13b shows the curves of *Mr* versus gradation parameter G/S under 15 different stress states (i.e., the 15 loading sequences). 

It can be seen that, at lower stress levels (e.g., the first to sixth loading sequences), the effect of gradation on *Mr* is not significant; however, at higher stress levels (e.g., the 7th to 15th loading sequences), the *Mr* increases by 11–23.5% with increasing G/S value (or relative content of coarse particles). It can be inferred from the above results that the relative content of coarse particles in recycled BDW aggregates may need to be increased appropriately when such materials are to be applied in pavement structures sustaining heavier axle loads; in other words, their gradations may need to be optimized. For pavement structures sustaining lower axle loads where recycled BDW aggregates are to be applied, the optimization of other material physical properties rather than gradation may be suitable.

## 4. Development of the Resilient Modulus Prediction Model

### 4.1. Evaluation of Existing Prediction Models of the Resilient Modulus

As a key input parameter of mechanistic-empirical pavement design and analysis, the resilient modulus is affected by the stress states and physical properties of materials, such as the moisture and density conditions, gradation, and particle shape. The resilient modulus of traditional unbound granular materials has been commonly predicted from purely empirical or mechanistic-empirical models [37]. A selection of the most commonly used ones is listed in Table 6, where models No. 1 to 3 are univariate models of stress states, and models No. 4 to 6 are bivariate models of stress states. 

In essence, such models were established from nonlinear regression analysis of laboratory testing results and are often not suitable for generalization to other conditions (e.g., material types, stress states, or physical properties) different from the original laboratory tests, i.e., the extrapolated applications of those statistical models could be erroneous and risky. The effect of particle breakage-induced gradation variations on the resilient modulus of recycled unbound aggregates has barely been considered in laboratory testing programs or prediction models. To address this drawback, this section presents the development of a resilient modulus prediction model from the previously mentioned laboratory RLT testing results to take into account gradation variations induced by particle breakage and degradation.

**Figure 14:**
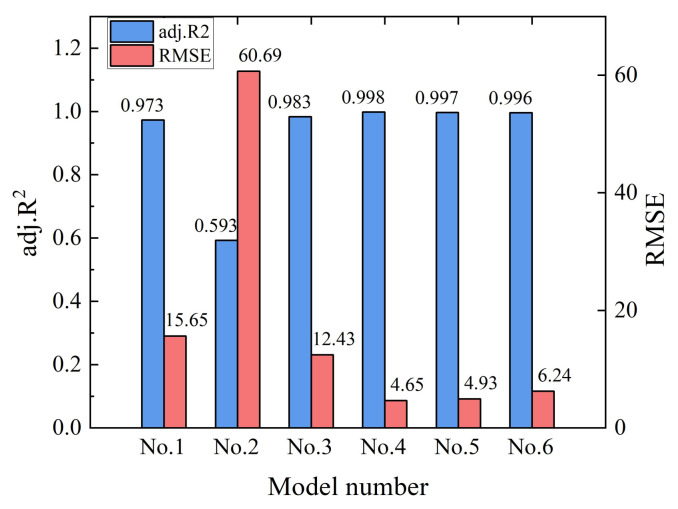
Goodness-of-fit index values of different resilient modulus prediction models for the gradation with G/S = 1.8.

To accomplish this, the six representative resilient modulus prediction models listed in Table 6 were fitted against laboratory RLT data obtained for different gradations and stress levels and described previously in Section 3.3, respectively. The goodness-of-fit indices of such different prediction models, e.g., the adjusted coefficient of determination (adj. R^2^) and root mean square error (RMSE), were calculated and comparatively analyzed as shown in Figure 14 for the gradation with G/S = 1.8. 

It can be seen that the adj. R^2^ value of the univariate prediction model No. 1 considering only the confining pressure was 0.973, which is much larger than that of 0.593 of the univariate prediction model No. 2 considering only deviator stress. This indicates that, compared to the deviatoric stress, the confining pressure is much more statistically significantly correlated with the resilient modulus of recycled BDW aggregates, which is consistent with the aforementioned laboratory testing results. The adj. R^2^ value of the univariate prediction model No. 3 considering only bulk stress was insignificantly improved when compared to model No. 1. 

The adj. R^2^ values of bivariate prediction models No. 4, No. 5, and No. 6, considering stress states, were all greater than 0.973 and showed insignificant differences from each other, which may be explained by the fact that all three prediction models include the effect of confining pressure. By considering the two variables of confining pressure and deviatoric stress separately, the prediction model No. 4 yielded an increased adj. R^2^ value of 0.998. Note that the octahedral shear stress is equivalent to deviatoric stress in conventional triaxial compression conditions where the minor principal stress *σ*_3_ and intermediate principal stress *σ*_2_ are equal; therefore, the prediction models No. 5 and No. 6 are essentially equivalent. 

Despite the effect of deviatoric stress being repeatedly considered in model No. 6 by the variables of bulk stress and octahedral shear stress, the goodness-of-fit was not improved compared to the model No. 5. In addition, the incorporation of standard atmospheric pressure (Pa) and the constant of 1 in the model No. 6 failed to improve the goodness-of-fit significantly. Therefore, in terms of the adj. R^2^ and RMSE values, the model No. 4 can be regarded as the best starting formulation to develop a new improved M_R_ prediction model for recycled BDW aggregates with breakage-induced gradation variation incorporated as an additional explanatory variable.

### 4.2. Development of New Improved Prediction Model

For the five different levels of gradation variations induced by particle breakage and degradation (i.e., five different G/S levels) of recycled BDW aggregates, the optimal model No. 4 selected from the six candidates (see Table 6) was then fitted against the aforementioned laboratory testing results of the resilient modulus. Table 7 summarizes the regressed model parameters k_1_, k_2_, and k_3_, as well as the calculated adj. R^2^ and RMSE values. It can be seen from Table 7 that the fitted model parameter k_1_ exhibited relatively greater differences among different gradations (or G/S values) than the model parameters k_2_ and k_3_. 

**Figure 15:**
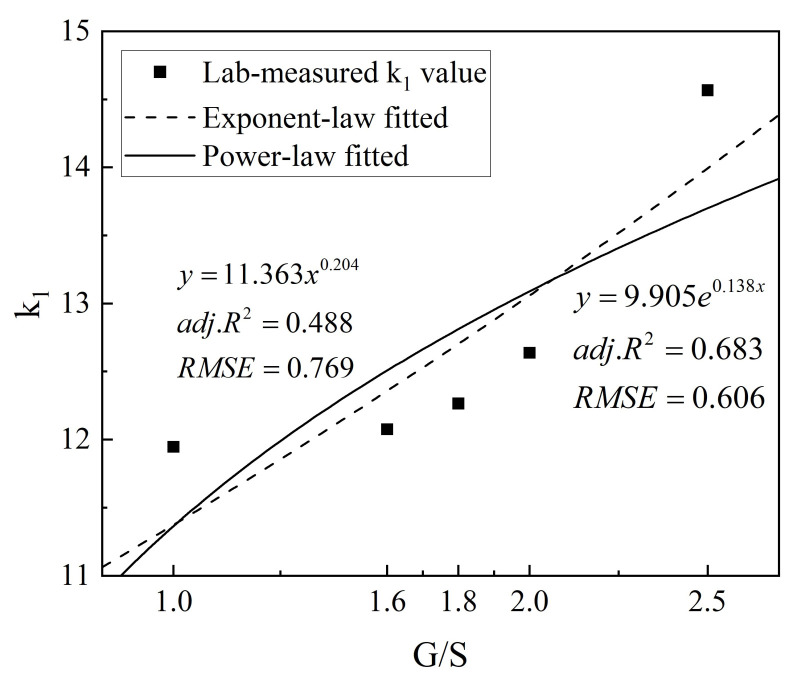
Fitting results of model parameter k_1_ against the G/S parameter.

Specifically, the model parameter k_1_ increased with increasing G/S level (or reduced level of breakage and degradation), while the model parameters k_2_ and k_3_ exhibited much fewer variations among different G/S levels (i.e., their values are distributed in relatively narrow ranges). In order to establish the relationship between the resilient modulus and particle-breakage-induced gradation variation, the regressed model parameter k_1_ of the model No. 4 was fitted against gradation parameter G/S by exponent- and power-law functions, respectively, as shown in Figure 15. 

According to the fitting results, the exponent-law model was used to relate the resilient modulus with the G/S parameter (or particle breakage index), and the additional gradation parameter G/S (or particle breakage index) was introduced into prediction model No. 4 to integrate the effects of stress states and breakage-induced gradation variation on the resilient modulus of recycled BDW aggregates. The new improved prediction model of the resilient modulus developed in this study is shown in Equation (6).

**Figure 16:**
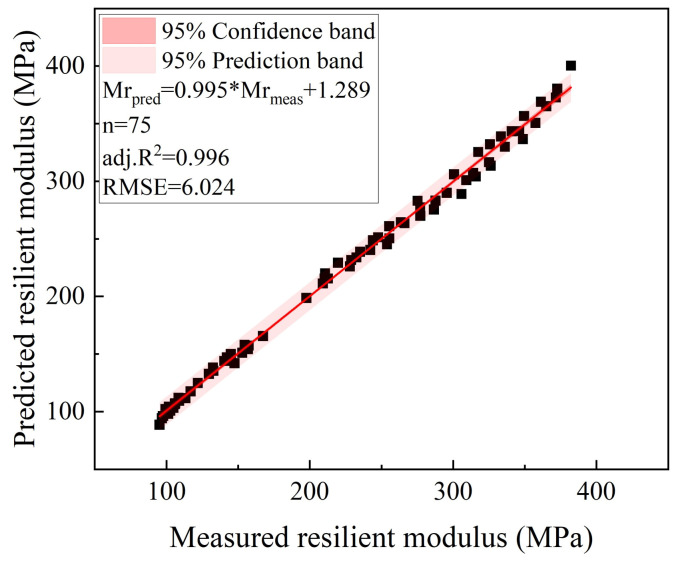
Comparison of the laboratory-measured resilient modulus values against those predicted from the new improved model (Equation (6)).

The resilient modulus values predicted by the new improved model (i.e., Equation (6)) for different gradations were compared against their laboratory-measured counterparts, as shown in Figure 16. From the adj. R^2^ and RMSE values, it can be indicated that the new improved model incorporating the exponential relation between MR and G/S can better predict the resilient modulus of recycled BDW aggregates with particle-breakage-induced gradation variations at varying stress states.

(6) $$M_{R}=k_{1}e^{k_{2}(G/S)}\sigma_{3}{}^{k_{3}}\sigma_{d}{}^{k_{4}}\;M_{R}=k_{1}e^{k_{2}(2.46e^{-0.023Br})}\sigma_{3}{}^{k_{3}}\sigma_{d}{}^{k_{4}}$$

## 5. Discussions

The application of recycled BDW aggregates in unbound pavement granular layers has been extensively studied. Recycled BDW particles commonly possess low-strength and highly breakable features, while loading-related particle breakage could lead to significant changes in the initial gradation and thus severely affect the hydromechanical properties, such as strength, deformation, and permeability. However, it can be seen from the above-mentioned literature survey that few previous studies have addressed particle breakage and the resulting gradation variations of recycled CDW aggregates as well as their possible consequences on the strength and resilient modulus behavior. 

This knowledge gap may hinder their reliable and widespread use in sustainable and long-lasting pavement construction. Therefore, in this study, five different gradations were designed to represent different levels of particle breakage (i.e., the breakage-induced gradation variations). The effects of particle breakage on the shear strength and resilient modulus properties of recycled BDW aggregates were investigated from laboratory monotonic triaxial compression tests and repeated-load triaxial tests.

According to the laboratory testing results, the apparent cohesion and internal friction angle of recycled BDW aggregates under consolidated drained conditions ranged from 35.3 to 57.5 kPa and from 30.2° to 54.3°, respectively. Such results are in reasonably well agreement with the cohesion value of 41 to 46 kPa and friction angle values of 49° to 51° reported by Arulrajah et al. [21,43] for the mixtures of recycled concrete aggregates, crushed bricks, and waste gravels. Therefore, it can be inferred that recycled BDW aggregates have the potential to substitute natural aggregates for use in unbound pavement granular base/subbase layers as they could meet the requirement of the minimum effective friction angle. 

In addition, both the deviator stress and confining pressure exhibited significant influences on the resilient modulus, while the effect of confining pressure was more pronounced. This is consistent with previous studies [44,45,46,47]. Accordingly, the stress- and breakage-dependent resilient modulus prediction model was developed based on the experimental data [45,47].

It can be seen from the monotonic triaxial compression test results that the apparent cohesion and internal friction angle also increased and decreased non-linearly with increasing relative content of fine particles, respectively. This indicates that particle breakage and degradation weaken the inter-particle frictional interactions among recycled BDW aggregates, thus, resulting in a reduction in the overall shear strength. In addition, the results from laboratory RLT tests revealed that the resilient modulus of recycled BDW aggregates gradually decreased with the increasing relative content of fine particles (or equivalently increasing particle breakage and degradation) at the same stress level. 

Overall, the test results show that the breakage-induced gradation variation, (i.e., the increase in the relative content of fine particles) weakened the shear strength and the ability to resist resilient and permanent deformation of recycled BDW aggregates subjected to the repeated applications of moving wheel loads. The findings could be useful for extended engineering applications of recycled BDW aggregates in unbound granular pavement base/subbase construction.

## 6. Summary and Conclusions

In view of the low-strength and breakage-susceptible nature of unbound aggregate particles recycled from building demolition waste (BDW), five different gradations were designed with the gravel-to-sand-ratio concept in this study to represent gradation variations induced by particle breakage and degradation. The shear strength and resilient modulus characteristics of the recycled BDW aggregates were investigated by conducting a series of laboratory monotonic triaxial compression tests and repeated-load triaxial (RLT) tests under different gradation and stress state conditions. The following major conclusions were drawn from the findings of this study.

- The results of particle morphology analysis showed that there appeared to be no significant differences in the particle morphology among different particle size groups of the same type of recycled BDW aggregates. Therefore, the effect of particle shape on the test results was excluded in this study by controlling the particle shape index to remain relatively similar or close to each other.
- The testing results of laboratory monotonic triaxial compression tests conducted under consolidated drained conditions on recycled BDW aggregates indicated that, as the relative content of fine particles increased, the apparent cohesion and internal friction angle showed a non-linear increase and decrease, respectively. This indicates that particle breakage and degradation weakened the inter-particle frictional interactions among recycled BDW aggregates, resulting in a reduction in the overall shear strength.
- The results from the laboratory RLT tests revealed that the resilient modulus of recycled BDW aggregates gradually increased with the decreasing relative content of fine particles or equivalently decreasing particle breakage and degradation) at the same stress level.
- Both the deviatoric stress and confining pressure exerted great influences on the *Mr* of recycled BDW aggregates, while a greater influence was exerted by the confining pressure when compared to the deviatoric stress.
- Based on the laboratory testing data, an improved resilient modulus prediction model that takes into account particle-breakage-induced gradation variation and stress states was proposed, and its better prediction accuracy was statistically confirmed.

In conclusion, this paper investigated the effects of breakage-induced gradation variation on the shear strength and resilient properties of recycled aggregates derived from building demolition waste (BDW) through a series of monotonic triaxial compression tests and repeated-load triaxial tests. We proposed a resilient-modulus prediction model incorporating particle breakage. The model provides a useful guide for predicting the resilient properties of recycled BDW aggregates with significant low-strength and a breakage-susceptible nature. In addition, considering the high water-absorbing potential of recycled BDW aggregates, further work is currently underway to investigate their water-sensitive properties for more a comprehensive understanding of the application of recycled BDW aggregates in pavement granular layers.

## Figures and Tables

**Table 1:** Percentages by weight of three different types of aggregate particles recycled from BDW (%).

Size	Gravel	Mortar	Brick	Others (Tiles, Wood, etc.)
5–10 mm	62.87	27.82	7.58	1.73
10–20 mm	68.49	25.75	3.88	1.88
20–40 mm	69.87	23.56	4.34	2.23

**Table 2:** The basic physical properties of aggregates recycled from BDW.

$\mathrm{Liquid}\;\mathrm{Limit}\;\omega_{L}/\%$	$\mathrm{Plastic}\;\mathrm{Limit}\;\omega_{P}/\%$	$\mathrm{Plasticity}\;\mathrm{Index}\;I_{P}/\%$	Crushing Value/%	Water Absorption/%	Specific Gravity
23.7~29.6	13.5~17.8	10.2~11.8	18.5~24.5	6.9~9.2	2.53~2.69

**Table 3:** The laboratory compaction test parameters as specified in the Chinese standard that was followed.

Test Method	Specimen Height (cm)	Specimen Volume (cm^3^)	Sub-Layers	Blows Per Sub-Layer	Maximum Particle Size (mm)
Heavy Ⅱ-2	12	2177	3	98	40

**Table 4:** Loading sequences of the resilient modulus tests of recycled BDW aggregates.

SequenceNo.	Confining Pressure, *σ*_3_/kPa	Contact Stress, *σ_c_*/kPa	Deviator Stress, *σ_d_*/kPa	Axial Stress, *σ_max_*/kPa	No. of Load Applications
0	103.4	10.3	93.1	103.4	1000
1	20.7	2.1	18.6	20.7	100
2	20.7	4.1	37.3	41.4	100
3	20.7	6.2	55.9	62.1	100
4	34.5	3.5	31	34.5	100
5	34.5	6.9	62	68.9	100
6	34.5	10.3	93.1	103.4	100
7	68.9	6.9	62	68.9	100
8	68.9	13.8	124.1	137.9	100
9	68.9	20.7	186.1	206.8	100
10	103.4	6.9	62	68.9	100
11	103.4	10.3	93.1	103.4	100
12	103.4	20.7	186.1	206.8	100
13	137.9	10.3	93.1	103.4	100
14	137.9	13.8	124.1	137.9	100
15	137.9	27.6	248.2	275.8	100

**Table 5:** The laboratory compaction and shear strength properties of recycled BDW aggregates with five different gradations.

Gradation Parameter G/S	Maximum Dry Density (g·cm^−3^)	Optimum Moisture Content (%)	Apparent Cohesion c’ (kPa)	Internal Friction Angle φ’ (°)
2.5	1.929	8.98	35.3	54.3
2.0	1.977	9.02	43.9	49.8
1.8	1.981	9.19	46.8	48.2
1.6	1.978	9.03	51.7	46.4
1.0	1.968	9.21	57.7	30.2

**Table 6:** Representative prediction models of the resilient modulus surveyed from the existing literature.

Model No.	Model Equations	Authors
No. 1	$M_{R}=k_{1}\sigma_{3}{}^{k_{2}}$	Monismith [38]
No. 2	$M_{R}=k_{1}\sigma_{d}{}^{k_{2}}$	Moossazadeh and Witczak [39]
No. 3	$M_{R}=k_{1}\theta^{k_{2}}$	Seed [40]
No. 4	$M_{R}=k_{1}\sigma_{3}{}^{k_{2}}\sigma_{d}{}^{k_{3}}$	Pezo [41]
No. 5	$M_{R}=k_{1}\theta^{k_{2}}\sigma_{d}{}^{k_{3}}$	Uzan [42]
No. 6	$M_{R}=k_{1}p_{a}{\left(\frac{\theta }{p_{a}}\right)}^{k_{2}}{\left(\frac{\tau_{oct}}{p_{a}}+1\right)}^{k_{3}}$	AASHTO 2004 [36]

$p_{a}$ is the standard atmospheric pressure and usually taken as 100 kPa; θ is the bulk stress, i.e., $\theta =\sigma_{1}+\sigma_{2}+\sigma_{3}$; $\tau_{oct}$ is the octahedral shear stress, i.e., $\tau_{oct}=\sqrt{{\left(\sigma_{1}-\sigma_{2}\right)}^{2}+{\left(\sigma_{1}-\sigma_{3}\right)}^{2}+{\left(\sigma_{2}-\sigma_{3}\right)}^{2}}/3$; and $k_{i}$ are the regression coefficients of different models.

**Table 7:** Regression parameters k_1_, k_2_, and k_3_ of resilient modulus prediction model No. 4 for different gradations.

G/S Value	k_1_	k_2_	k_3_	adj. R^2^	RMSE
1.0	11.946	0.544	0.119	0.996	5.078
1.6	11.076	0.559	0.128	0.996	5.559
1.8	12.265	0.550	0.127	0.998	4.654
2.0	12.638	0.564	0.112	0.993	8.349
2.5	14.567	0.550	0.114	0.995	6.943

## Data Availability

Not applicable.
